# 2018 Korean society of hypertension guidelines for the management of hypertension: part III-hypertension in special situations

**DOI:** 10.1186/s40885-019-0123-y

**Published:** 2019-08-01

**Authors:** Kwang-il Kim, Sang-Hyun Ihm, Gheun-Ho Kim, Hyeon Chang Kim, Ju Han Kim, Hae-Young Lee, Jang Hoon Lee, Jong-Moo Park, Sungha Park, Wook Bum Pyun, Jinho Shin, Shung Chull Chae

**Affiliations:** 1 Department of Internal Medicine, Seoul National University Bundang Hospital, Seoul National University College of Medicine, Seongnam, Korea; 2 Department of Internal Medicine, College of Medicine, The Catholic University of Korea, Seoul, Korea; 3 Department of Internal Medicine, Hanyang University College of Medicine, Seoul, Korea; 4 Department of Preventive Medicine, Yonsei University College of Medicine, Seoul, Korea; 5 Department of Internal Medicine, School of Medicine, Chonnam University, GwangJu, Korea; 6 Department of Internal Medicine, Seoul National University College of Medicine, Seoul, Korea; 7 Department of Internal Medicine, Kyungpook National University, School of Medicine, 130 Dongdeok-ro, Jung-gu, Daegu, Korea; 8 Department of Neurology, Nowon Eulji Medical Center, Eulji University, Seoul, Korea; 9 Department of Internal Medicine Division of Cardiology, Yonsei University College of Medicine, Seoul, Korea; 10 Department of Internal Medicine, School of Medicine, Ewha Womans University, Seoul, Korea

**Keywords:** Antihypertensive treatment, Blood pressure, Cardiovascular complications, Cardiovascular risk, Guidelines, Hypertension

## Abstract

Treatment of hypertension improves cardiovascular, renal, and cerebrovascular outcomes. However, the benefit of treatment may be different according to the patients’ characteristics. Additionally, the target blood pressure or initial drug choice should be customized according to the special conditions of the hypertensive patients. In this part III, we reviewed previous data and presented recommendations for some special populations such as diabetes mellitus, chronic kidney disease, elderly people, and cardio-cerebrovascular disease.

## Hypertension in special situations

### White coat hypertension and masked hypertension

**Table:** 

Recommendations	Class	Level	References
It is reasonable to exclude the presence of white coat hypertension (HTN) by using either ambulatory blood pressure monitoring (ABPM) or home blood pressure monitoring (HBPM) before initiation of antihypertensive medication.	**I**	**C**	[1]
It is reasonable to check ABPM or HBPM before change of antihypertensive drug treatment intensification.	**IIa**	**C**	[2]
It is reasonable to check ABPM or HBPM for the adults with prehypertension or suspected of masked HTN	**IIa**	**B**	[3]
For the patients with masked HTN, lifestyle modifications and antihypertensive drug therapy may be reasonable.	**IIa**	**C**	[4]

It is important to detect white coat HTN [high office blood pressure (BP) but normal out-of-office BP] and masked HTN (normal office BP but high out-of-office BP) using out-of-office BP measurement. White coat effect is more frequently observed in women and older adults. If the diagnosis is not made correctly, it may result in side effects associated with unnecessary medication. Therefore, it is recommended to exclude white coat HTN as much as possible before initiating antihypertensive medication. In patients who are already on medication, it is important to check the effectiveness before adding medication for more aggressive treatment.

For the patients with white coat HTN, lifestyle modifications and regular BP monitoring are recommended. Although there is little evidence, pharmacological treatment as well as lifestyle modifications could be considered when metabolic disturbances and/or subclinical organ damage occurs with white coat HTN [5]. Pharmacological treatment for masked HTN may be beneficial because it has shown a similar cardiovascular (CV) risk profile as sustained HTN [4, 6, 7]. Masked HTN has a higher risk of cardiovascular disease (CVD), thus thorough treatment is considered. There are no clear criteria for the diagnosis of masked HTN and the threshold or reproducibility [8, 9]. However, masked HTN is more common in smokers or in patients with work-related stress and it is observed in about 30% among patients with prehypertension. Special attention should be paid to the patients with metabolic syndrome with high cardiovascular risk, target organ damage, diabetes mellitus (DM), chronic kidney disease (CKD), smokers, heavy drinking, exercise induced hypertension, and occupational stress for the diagnosis and treatment of masked HTN.

### Metabolic syndrome

**Table:** 

Recommendations	Class	Level	References
Lifestyle modifications such as diet, weight reduction, and exercise are recommended for the hypertensive patients with metabolic syndrome.	**I**	**B**	[10, 11]
Angiotensin converting enzyme (ACE) inhibitors/angiotensin II receptor blockers (ARBs) or calcium channel blockers can be considered as the antihypertensive agents for patients with metabolic syndrome.	**IIa**	**C**	
Antihypertensive medication is not recommended for prehypertensive patients with metabolic syndrome.	**III**	**A**	[12–14]

Many hypertensive patients have obesity and metabolic abnormalities with alterations of lipid and glucose metabolism. Furthermore, subclinical organ damage is common in these patients. Metabolic syndrome consists of abdominal obesity, dyslipidemia, dysglycemia, and raised BP. The criteria for clinical diagnosis of metabolic syndrome are: 1) abdominal obesity; 2) fasting glucose ≥100 mg/dL (diabetes included); 3) triglycerides ≥150 mg/dL; 4) high-density lipoprotein (HDL)-cholesterol < 40 mg/dL in men and < 50 mg/dL in women; and 5) BP ≥130/85 mmHg [15]. Presence of three or more of these criteria confirms the diagnosis of metabolic syndrome. Abdominal obesity is usually estimated by the measurement of waist circumference. However, cut-off points of waist circumference for abdominal obesity in Korean adults are not established. Cut-off points of waist circumference, which are commonly used, are 1) ≥90 cm in men; ≥80 cm in women (International Obesity Task Force criteria for Asian-Pacific population); and 2) ≥90 cm in men; ≥85 cm in women (Korean adult specific values) [15, 16].

The prevalence of metabolic syndrome has been increased over the last 10 years, as reported in the Korean National Health and Nutritional Survey. The prevalence of metabolic syndrome in Korean aged over 30 years is 30.5% [17]. However, the prevalence of metabolic syndrome is twice as high among the hypertensive patients [18].

In Western countries, people with metabolic syndrome have at 1.5–2-fold higher risk of CV events and death than those without metabolic syndrome and incident diabetes is 5-fold higher in people with metabolic syndrome [19, 20].

In Asian countries, metabolic syndrome had a relative risk of incident diabetes of 3–4, which is a little lower compared to that in Western countries [21, 22]. The most powerful predictor of incident diabetes is hyperglycemia in people with metabolic syndrome. However, metabolic syndrome, even without hyperglycemia, was associated with an increased risk of incident diabetes; the relative risk is 2.4 in Japanese population [22]. In addition to metabolic syndrome, HTN is associated with twice higher risk of incident diabetes [23, 24].

In hypertensive patients with metabolic syndrome, antihypertensive treatment is used to prevent CV morbidity and mortality, while lowering or preventing incident diabetes. Antihypertensive treatment in non-diabetic patients with metabolic syndrome is discussed below, while that treatment for diabetic/CVD patients with metabolic syndrome is discussed in other respective sections of the special situations chapter. Lifestyle modifications, especially weight reduction and regular exercise, are strongly recommended in all hypertensive patients, as they decrease BP, improve metabolic abnormalities, and delay incident diabetes [10, 11].

Hypertensive patients should be treated with antihypertensive medication as well as lifestyle modification, but there is no evidence available for the drug treatment in prehypertensive patients [12–14].

Antihypertensive drugs should lower BP effectively as well as have favorable or neutral effects on insulin sensitivity and metabolic abnormalities. Thus, ACE inhibitors, ARBs, and calcium channel blockers are preferred. Among beta-blockers, vasodilating beta-blockers such as carvedilol and nebivolol can be used as combination therapy with ACE inhibitors/ARBs.

Thiazides and thiazide-like diuretics are avoided as monotherapy or at high doses, but are used as combination therapy or at low doses. High dose diuretics can induce hypokalemia and new onset diabetes, and have unfavorable effects on lipid metabolism. The preferred approach is combination of ACE inhibitors/ARBs with diuretics to minimize their unfavorable effects on glucose and lipid metabolism.

### Diabetes mellitus

**Table:** 

Recommendations	Class	Level	References
In patients with DM and HTN, systolic BP should be less than 140 mmHg.	**I**	**A**	[25–29]
In patients with DM and HTN, diastolic BP should be less than 85 mmHg.	**I**	**B**	[30–32]
In patients with DM and CVD, BP should be less than 130/80 mmHg.	**IIa**	**C**	[33]
In hypertensive patients with DM, all hypertensive drugs are recommended as first-line antihypertensive agents.	**I**	**A**	[27, 32]
ACE inhibitors or ARBs are recommended for patients with microalbuminuria or proteinuria.	**I**	**B**	[34–36]

The prevalence of HTN is two-fold in diabetic patients compared to that in the general population, and the occurrence of diabetes is 2.5-fold higher in hypertensive patients [37, 38]. The coexistence of HTN and diabetes causes the progression of CVD, stroke, and renal disease. The high risk of HTN in diabetic patients is related with weight gain, hyperinsulinemia, sympathetic hyperactivity, and salt retention. Additionally, hyperglycemia itself can further increase the risk of HTN by increasing arterial stiffness and progressing atherosclerosis. The nocturnal dipping disappears in diabetic patients, and it is associated with subclinical organ damage such as left ventricular hypertrophy (LVH) and microalbuminuria. In the UK Prospective Diabetes Study (UKPDS)-36, each 10 mmHg decrease in mean systolic blood pressure (SBP) was associated with risk reductions of 12% for any complication related to diabetes, 15% for deaths related to diabetes, 11% for myocardial infarction, and 13% for microvascular complications [39]. Previous studies have shown that appropriate BP control can reduce the incidence of CVD [34, 40–43].

The recommended target for BP in diabetic patients is < 140/85 mmHg. In previous guidelines, the recommended BP was < 130/80 mmHg or < 140/80 mmHg in diabetic patients [34, 40–43]. However, recent studies have failed to show any beneficial effect on CV outcome with strict BP control [28, 44]. Due to the beneficial effect of strict BP control in Systolic Blood Pressure Interventional Trial (SPRINT)-eligible patients among Action to Control Cardiovascular Risk in Diabetes (ACCORD) study, BP should be lowered less than 130/80 mmHg in patients with diabetes and CVD [33].

According to a recent meta-analysis, all classes of antihypertensive agents, such as ACE inhibitors, ARBs, calcium channel blockers, beta-blockers and diuretics can be used in diabetic patients [31]. However, ACE inhibitors/ARBs are recommended as first-line antihypertensive therapy in patients with microalbuminuria or proteinuria [32, 34–36]. The superiority of one antihypertensive class over others is controversial. The choice of a particular class is less significant in practice because two or more antihypertensives should be combined in order to obtain sufficient decrease in BP in most diabetic patients. However, the combination of beta-blockers and thiazide diuretics is not recommended as it could worsen glucose control by increasing insulin resistance. Sodium-glucose co-transporter-2 (SGLT-2) inhibitor can decreased BP, thus it should be used cautiously with antihypertensive medication [45–47].

### Hypertension in older adults

**Table:** 

Recommendations	Class	Level	References
SBP goal of less than 140 mmHg is recommended for noninstitutionalized, ambulatory community-dwelling adults (≥65 years of age).	**IIa**	**B**	[48, 49]

The treatment of HTN in older adults reduces the risk of CVD and mortality. The benefit of treatment is also observed with regard to isolated systolic HTN. Accordingly, HTN needs to be actively diagnosed and treated in older adults.

However, the benefit of pharmacological treatment for grade I hypertensive patients older than 80 years remains controversial. Thus, the characteristics of the patient should be considered. The characteristic findings of elderly hypertensive patients are increased SBP and widened pulse pressure due to increased central arterial stiffness. Moreover, atherosclerotic renovascular HTN is commonly observed. Non-dipper, increased daytime BP variability, and orthostatic or postprandial hypotension are also characteristic findings in elderly patients with HTN.

The non-pharmacological treatment in elderly HTN patients is effective; however, the impact on the patient’s quality of life should be considered [50]. Target SBP for older patients is < 140 mmHg, but orthostatic hypotension should be avoided [51]. Additional studies are required to confirm the target SBP among the frail older patients. The initial dose of the pharmacological treatment is reduced by half in younger patients, and gradually increased. Elderly hypertensive patients without co-morbidities should be treated with ACE inhibitors, ARBs, calcium channel blockers, and diuretics [52–55]. Beta-blockers do not improve prognosis as much as other drug classes in elderly hypertensive patients. However, beta-blockers would be effective in patients with angina, heart failure, or tachycardia. Combination therapy with two or more drugs should be considered if the BP is not controlled with monotherapy. Patients with co-morbidities require special consideration. It is safe to slowly lower the BP in elderly patients. Complications caused by medication should be monitored when increasing the drug dose. Orthostatic hypotension should be periodically checked by positional BP measurement.

### Cardiac diseases

#### Coronary artery disease

**Table:** 

Recommendations	Class	Level	References
In adults with coronary artery disease (CAD) and HTN, a BP target of 130/80 mmHg is recommended.	**IIa**	**B**	[56–60]

HTN is a major risk factor of CAD and is associated with the occurrence of myocardial infarction. The incidence of ischemic heart disease increases when SBP is > 140 mmHg and mortality increases when SBP is > 120 mmHg [61, 62]. Considering J-curve phenomenon, it is important not to decrease DBP < 70 mmHg, especially in older patients, multivessel disease without revascularization, and diabetic patients. The preferred drugs within 1 month after acute myocardial infarction are beta-blockers and ACE inhibitors [63, 64]. Any first line antihypertensive drugs are available in patients with ischemic heart disease, but beta-blockers and calcium channel blockers are initially considered for the symptomatic CAD.

#### Chronic heart failure

**Table:** 

Recommendations	Class	Level	References
In adults with HTN and increased risk of heart failure, a BP target of 130/80 mmHg is recommended.	**I**	**B**	[65]
Adults with heart failure with reduced ejection fraction (HFrEF) and hypertension should be prescribed to attain a BP of 130/80 mmHg.	**I**	**B**	[66]

HTN is the most important risk factor in heart failure [67–69]. Strict BP control in hypertensive patients with HFrEF is helpful for the prevention of heart failure aggravation and CV events as well as mortality reduction [70]. Most BP-lowering drugs such as diuretics, beta-blockers, ACE inhibitors, and ARBs are effective in the prevention of heart failure. In decompensated heart failure patients, a 25% reduction of BP can be achieved with vasodilator and loop diuretics within few hours. For the HFrEF and hypertensive patients, beta-blockers, ACE inhibitors, ARBs, and aldosterone antagonists are recommended considering the beneficial effects on mortality reduction and re-hospitalization. Thiazides can be used for the additional BP lowering. A dihydropyridine calcium channel blocker can be used, but in contrast, a non-dihydropyridine calcium channel blocker such as verapamil or diltiazem should not be used because of negative inotropic effects [71]. HTN is also a risk factor for heart failure with preserved ejection fraction (HFpEF) and it is likely that strict BP control is helpful for the CV events and mortality reduction in HFpEF patients.

#### Atrial fibrillation

Hypertension is one of the risk factor for atrial fibrillation and atrial fibrillation is frequently observed in hypertensive patients, but it can be prevented with BP control [72, 73]. Patients with HTN and atrial fibrillation have a high risk of thromboembolism and need chronic antithrombotic treatment if no contraindications are present [74]. Recently, antithrombotics such as direct thrombin inhibitors (dabigatran) and factor Xa inhibitors (rivaroxaban, apixaban, edoxaban) have been shown to be effective and relatively safe compared with the classic antithrombotic therapy using warfarin [75, 76]. In patients with atrial fibrillation and HTN, lowering the BP can decrease the incidence of fatal bleeding during antithrombotic treatment. However, BP should be maintained above 120/70 mmHg [77, 78].

### Vascular diseases

#### Carotid atherosclerosis

The progression of carotid atherosclerosis can be prevented by lowering BP. For this purpose, calcium channel blockers and ACE inhibitors are superior to beta-blockers and diuretics [79, 80].

#### Arterial stiffness

Most antihypertensive drugs decrease vascular stiffness because BP reduction can decrease vascular wall stress and pulse wave velocity. However, it is not clear whether there is any difference in the reduction of arterial stiffness according to drug class [81–83]. Renin-angiotensin-aldosterone inhibitors can decrease the pulse wave velocity, independent of BP reduction [84–86]. Vasodilating beta-blockers are better for central aortic SBP reduction compared to atenolol. Although an improvement in vascular stiffness with antihypertensive drugs has been reported in numerous studies, it is still uncertain whether the improvement in vascular stiffness is closely related to the CV benefits, except for CKD patients [87]. Further studies are needed to determine the relationship between vascular stiffness and CV outcomes.

#### Peripheral arterial disease

It is important to control the CVD risk factors because patients with peripheral arterial disease (PAD) have a higher risk of CV mortality (10-year mortality of 40%) [88]. Lowering the SBP decreases the leg amputation rate and mortality in hypertensive patients with diabetes and PAD [39]. The target BP is around 130/80 mmHg in patients with PAD according to a recent meta-analysis and the SPRINT trial [89–91].

Lifestyle modifications such as salt restriction, weight control, moderation of alcohol intake, and regular aerobic exercise are very important. Pharmacological treatment consists of ACE inhibitors, ARBs, and aspirin [92, 93]. ACE inhibitors decrease long-term CV events from either BP lowering effects or indirect BP lowering effects [41, 94]. However, other drugs are also effective in the reduction of CV events associated with BP lowering [90, 94–97]. Furthermore, it is important to evaluate and manage CV risk factors other than HTN, such as lipid and blood glucose. The appropriate drugs are determined according to the presence of heart failure or coronary artery disease. In general, beta-blockers are relatively contraindicated to avoid worsening of PAD symptoms. However, some reports have revealed that beta-blockers do not aggravate the symptoms in patients with mild to moderate PAD Therefore, beta-blockers are effective in PAD patients with co-existing ischemic heart disease or tachycardia [98, 99]. Renal artery stenosis is frequently observed in patients with HTN and PAD. Overall, ongoing evaluation of the disease and monitoring are needed during HTN treatment [100].

#### Aortic disease

In patients with aortic aneurysm, it is strongly recommended to lower the BP to the lowest levels tolerated by the patient [101]. Beta-blockers are preferred owing to their ability to reduce the maximum ventricular ejection of the left ventricle, as well as the BP and heart rate. In acute aortic syndrome, including aortic dissection, the BP and heart rate should be controlled aggressively with antihypertensive medication including beta-blockers. BP should be lower less than 140 mmHg within 1 h and maintained less than 120 mmHg thereafter. It is recommended to reduce BP around 130/80 mmHg in patients with atherosclerosis [60, 91].

### Chronic kidney disease

**Table:** 

Recommendations	Class	Level	References
In adults with HTN and CKD (with albuminuria ≥30 mg/day, or albumin-to-creatinine ratio ≥ 30 mg/g), treatment with ACE inhibitor or ARB may be reasonable.	**IIa**	**B**	[30, 102–104]

CKD is defined as the presence of kidney injury for > 3 months, with markers of kidney injury being a decrease in estimated glomerular filtration rate (eGFR) (< 60 mL/min/1.73 m^2^), urinary abnormalities including albuminuria (> 30 mg/day or albumin-to-creatinine ratio > 30 mg/g), hematuria and pyuria, electrolyte disturbances caused by tubular dysfunction, renal structural abnormalities detected by imaging or biopsy procedures, and renal transplants. CKD patients frequently suffer from HTN; hence, the rate of decline in renal function and the incidence of CV complications can be reduced with HTN control [105, 106]. However, we still need to determine the target BP levels, optimal tools to be used in HTN control, and the real benefits and risks associated with treatment [107].

The recent 2017 American College of Cardiology/American Heart Association/American Society of Hypertension guideline recommended a BP target of < 130/80 mmHg in all CKD patients [108]. However, previous major clinical trials failed to show any beneficial effect of strict BP control in non-proteinuric CKD patients. Accordingly, CKD patients without albuminuria are recommended to maintain BP < 140/90 mmHg [109–111]. On the other hand, randomized controlled trials have suggested that a lower target may be beneficial in proteinuric CKD patients. Thus, we recommend that CKD patients with albuminuria should be treated to maintain SBP < 130 mmHg [30, 112–114]. BP target levels do not depend on the presence of DM.

Lifestyle modifications should be used as a basic tool for BP control in all hypertensive CKD patients. Although no large scale randomized controlled trials have reported the effects of lifestyle modification on clinical outcomes in CKD patients, the beneficial effects can be inferred from the results reported in previous studies in general populations [115–121]. The 2011–2014 Korea National Health and Nutrition Examination Survey showed that body mass index (BMI) associated with the risk of CKD [122]. We recommend achieving or maintaining a healthy weight (BMI 20 to 25), lowering salt intake to < 90 mmol (< 2 g) per day of sodium unless contraindicated, undertaking regular exercise compatible with CV health and tolerance, limiting alcohol intake to < 2 standard drinks per day for men and < 1 standard drink per day for women.

Pharmacological treatment in CKD patients includes single or multiple antihypertensive therapies to achieve the target BP. Although any antihypertensive can be used in CKD patients, ACE inhibitors or ARBs have been reported to be renoprotective owing to the reduction in albuminuria and improvement in the rate of decline of the glomerular filtration rate [30, 102–104]. Thus, we recommend that ACE inhibitors or ARBs should be used in CKD patients with albuminuria. ACE inhibitors or ARBs are preferred in both diabetic and non-diabetic CKD patients with either microalbuminuria (30–300 mg/day) or macroalbuminuria (> 300 mg/day). However, the combination of ACEIs and ARBs in CKD patients are not recommended.

It should be noted that there are occasions when the BP target and the preferred agent mentioned above may be inappropriate. Treatment should be individualized based on the patient’s age, presence of albuminuria and comorbidities. Diabetic or elderly patients need to be questioned about orthostatic dizziness because of the possibility of postural hypotension [123–125]. ACE inhibitors and ARBs are contraindicated in bilateral renal artery stenosis, and should be used with caution in patients with diffuse atherosclerosis.

### Cerebrovascular diseases

The risk of ischemic and hemorrhagic stroke increases proportionally as the BP increases, with HTN being the most common modifiable risk factor in stroke prevention and having the highest attributable risk for stroke in the population. HTN treatment, particularly SBP control, will remarkably reduce the incidence of stroke. For the management of high BP, lifestyle modifications (weight loss, low-fat diet, reduced salt intake, exercise or physical activity, moderation of alcohol intake, and smoking cessation) must routinely precede drug therapy. According to an epidemiologic study, with each increase of 20/10 mmHg from BP level of 115/75 mmHg or higher, deaths from stroke increased at least two-fold. Conversely, a 10/5 mmHg decrease in BP resulted in a 40% decrease in deaths from stroke [61]. In addition, a meta-analysis of clinical studies showed that stroke risk was expected to decrease by about 30–40% by lowering the BP by 10/5 mmHg with drug therapy, regardless of the past history of the patient [63, 126, 127]. For the primary prevention of stroke, it is recommended to maintain the BP < 140/90 mmHg. Although it remains unknown whether a specific drug or class is superior to other antihypertensive drugs in the prevention of stroke, a limited number of reports have shown that calcium channel blockers, ACE inhibitors, or ARBs are superior to beta-blockers [128]. However, for the primary prevention of stroke, it is most important to lower the BP based on an individualized approach for each patient rather than the choice of a specific drug or drug class [129].

#### Acute ischemic stroke

**Table:** 

Recommendations	Class	Level	References
If blood pressure is high in patients with acute ischemic stroke suitable for intravenous thrombolytic therapy, we recommend lowering the BP to less than 185/110 mmHg before initiating intravenous thrombolytic therapy.	**I**	**B**	[130, 131]
BP in patients with acute ischemic stroke should be reduced to 185/110 mmHg or lower before intravenous thrombolytic therapy and maintained below 180/105 mmHg for 24 h.	**I**	**B**	[132]
Starting or resuming anti-hypertensive medication during hospitalization in ischemic stroke patients with BP greater than 140/90 mmHg who are neurologically stable is safe and reasonable to improve long-term BP control, unless contraindicated.	**IIa**	**B**	[133, 134]
In patients with BP of 220/120 mmHg or higher who did not receive intravenous thrombolysis or thrombectomy, and have no comorbid conditions requiring acute antihypertensive treatment, the benefit of initiating or reinitiating treatment of HTN within the first 48 to 72 h is uncertain. It might be reasonable to lower BP by 15% during the first 24 h after onset of stroke	**IIb**	**C**	[135]
In patients with BP less than 220/120 mmHg who did not receive intravenous thrombolysis or endovascular treatment and do not have a comorbid condition requiring acute antihypertensive treatment, treatment of HTN within the first 48 to 72 h after an acute ischemic stroke is not effective to prevent death or dependency.	**III**	**A**	[133, 134, 136]

In general, the BP rises in acute ischemic stroke [137]. It is assumed that BP increases owing to the acute stress, previous HTN, and the automatic compensation in an attempt to maintain perfusion of the brain tissue in the ischemic state. Thus, continuous BP monitoring is important, as a sudden drop in BP should be avoided to maintain the appropriate perfusion to the brain. Although a previous study has shown that ARB administration for 1 week in stroke patients within a week of the attack reduced the mortality after a 12-month period, large-scale studies are needed [41, 138]. Conversely, because active treatment against rising BP would reduce the perfusion to the ischemic area and expand the area of the infarction, it is undesirable to lower the BP actively within the period of 1 week of the acute ischemic stroke [7, 139].

When thrombolytic therapy is used in the hyperacute period of an ischemic stroke, the incidence of bleeding is closely related to the BP before and after thrombolysis; hence, the target BP should be < 185/110 mmHg. For thrombolytic therapy using tissue-plasminogen activator (t-PA), the drug could only be administered after the BP was < 185/110 mmHg. The antihypertensive regimens consisting of intravenous drugs such as labetalol, nicardipine, diltiazem, nitroglycerin, and nitroprusside are recommended [140–143]. In the acute phase of the ischemic stroke, it is recommended to use antihypertensive drugs only when BP is > 220/120 mmHg, to avoid a decrease in the cerebral perfusion around the infarct area [144]. Target BP levels should be 85–90% of the baseline BP. However, in cases of hypertensive encephalopathy, aortic dissection, acute renal failure, acute pulmonary edema, and acute myocardial infarction, it is recommended to lower the BP sufficiently in order to prevent complications associated with the elevated BP itself [140, 141].

#### Acute parenchymal hemorrhage

**Table:** 

Recommendations	Class	Level	References
If the SBP of a patient with acute parenchymal hemorrhage within 6 h of the onset is 150–220 mmHg, immediate lowering of SBP may be considered with a target SBP above 140 mmHg.	**IIb**	**A**	[145, 146]
In adults with intracerebral hemorrhage who present with SBP greater than 220 mmHg, it is reasonable to use continuous intravenous drug infusion and close BP monitoring to lower SBP.	**IIa**	**C**	[147–149]

From a theoretical viewpoint, the optimal BP treatment in the acute phase of a parenchymal hemorrhage could prevent re-bleeding and subsequent expansion of a hematoma and edema; hence, it is recommended to lower the BP during the acute phase of the hemorrhage. If the SBP is ≥200 mmHg or if the mean BP is ≥150 mmHg, the BP should be lowered by monitoring BP every 5 min. In patients with increased intracranial pressure, BP should be lowered by maintaining the cerebral perfusion pressure between 60 and 80 mmHg using an intracranial pressure monitoring device only when the SBP is > 180 mmHg or the mean BP is > 30 mmHg. Because a sudden drop of BP during the acute phase is associated with higher mortality rates, it is recommended to maintain a cerebral perfusion pressure of ≥60 mmHg. When the SBP is 180 mmHg or the mean BP is 130 mmHg—as long as there is no evidence of increased intracranial pressure, with the assessment of BP every 15 min—the BP should be lowered below the level of 80% of baseline BP, < 160/90 mmHg, or mean BP < 110 mmHg. Antihypertensive regimens including intravenous drugs such as labetalol, nicardipine, diltiazem, nitroglycerin, and nitroprusside are recommended [150]. Despite recent studies showing benefits associated with lowering BP to less than 140 mmHg during the acute phase, the supportive evidence is insufficient; hence, the BP should not be reduced to below 140 mmHg [145, 146, 151].

#### Secondary prevention of stroke

**Table:** 

Recommendations	Class	Level	References
Patients with previously treated HTN who experience stroke or transient ischemic attack should be advised to resume HTN medication a few days after stroke for the prevention of recurrent stroke or vascular disease.	**I**	**A**	[76, 152, 153]
We recommend thiazide diuretic, ACE inhibitor/ARB or combination treatment consisting of a thiazide diuretic plus ACE inhibitor/ARB for the treatment of HTN in stroke or transient ischemic stroke patients.	**I**	**A**	[76, 152, 154]
It is reasonable to consider calcium channel blockers to control HTN in stroke or transient ischemic stroke patients.	**IIa**	**C**	[155, 156]
Patients without HTN treatment are advised to start HTN treatment several days after stroke or transient ischemic attack to prevent stroke and vascular disease recurrence if BP is above 140/90 mmHg.	**I**	**B**	[152–154]
The usefulness of antihypertensive treatment has not been established when blood pressure is below 140/90 mmHg after stroke or transient ischemic attack.	**IIb**	**C**	[157]
Patients with lacunar infarction, a BP goal of less than 130/80 mmHg may be reasonable.	**IIb**	**B**	[158]

HTN treatment as a measure of secondary prevention after stroke significantly reduces mortality and the recurrence of stroke or vascular diseases [126, 154, 159, 160]. Regardless of the history of HTN, treatment of HTN after stroke significantly reduces the mortality and complications associated with HTN. Lifestyle modifications should be maintained in addition to the pharmacological treatment. For the selection of the optimal antihypertensive drugs, the individual characteristics of the patient such as the presence of extracranial cerebrovascular disease, kidney disease, heart disease, and diabetes should be considered. In a recent meta-analysis, a combination therapy using ACE inhibitors and diuretics is preferred [154].

### Erectile dysfunction

Erectile dysfunction is considered to be one of the a CV risk factors associated with poor prognosis [161]. Therefore, risk factors such as DM, dyslipidemia, and smoking should be controlled aggressively, and lifestyle modifications should be recommended to the patients with erectile dysfunction to reduce the CV risk. However, most cases of the erectile dysfunction are not properly diagnosed by doctors and only a minority of patients seeks medical advice. Therefore, more cautious history taking and comprehensive management are required for the improvement of CV prognosis among hypertensive patients with erectile dysfunction [162].

In general, the prevalence of erectile dysfunction associated with antihypertensive medication is reported to be 0–25%, but the prevalence may be affected by underlying conditions such as endothelial dysfunction, and oxidative stress [163]. Erectile dysfunction related to antihypertensive medication is usually observed within 4 weeks. Beta-blockers and diuretics are known to cause erectile dysfunction, and ACE inhibitors and calcium channel blockers are neutral, whereas ARBs are reported to be beneficial [164]. A vasodilating beta-blocker such as carvedilol, nebivolol, and labetalol may be an alternative for hypertensive patients with erectile dysfunction. A phosphodiesterase-5 (PDE5) inhibitor is relatively safe and effective in patients with erectile dysfunction caused by antihypertensive medications, and the additional BP lowering effect is negligible. However, the BP after administration of PDE5 inhibitors should be closely monitored and PDE5 inhibitors must not be co-administered with nitrates [165, 166].

### Pregnancy

High BP occurring during pregnancy can be divided into four categories: 1) chronic HTN in pregnancy: existing HTN or taking antihypertensive medication before the 20th week of pregnancy; 2) gestational HTN: newly diagnosed HTN after the 20th week of pregnancy in the absence of proteinuria; 3) pre-eclampsia: HTN diagnosed after 20 weeks of pregnancy accompanied by proteinuria (albumin more than 300 mg in 24 h urine or urine albumin/creatinine ratio of 300 mg/g or greater); and 4) pre-eclampsia superimposed on chronic HTN: pre-eclampsia diagnosed in the chronic HTN pregnant women. Based on the BP level, it is classified as mild: 140–149/90–99 mmHg, moderate: 150–159/100–109 mmHg, and severe: 160/110 mmHg or higher.

Generally, there are few controversies regarding drug treatment for BP of > 160/110 mmHg or higher. Previous studies have reported that patients with BP > 150/95 mmHg are more likely to be hospitalized due to incident stroke during the peripartum period [167, 168]. In pregnancy, BP should be controlled below 150/100 mmHg, but it is not recommended to lower DBP below 80 mmHg [169, 170].

Antihypertensive drugs used during pregnancy are methyldopa, labetalol, and nifedipine. Specific drugs are selected in consideration of the drug class previously used, their side effects, and the risk of teratogenicity. Beta-blockers can cause fetal growth retardation, thus they can be used later in the pregnancy. Diuretics should be prescribed cautiously because they can induce volume depletion. ACE inhibitors or ARBs may increase the risk of congenital malformations in pregnancy, it is recommended to replace these drugs before pregnancy or when planning a pregnancy. If a pregnancy is detected during the administration of ACE inhibitors or ARBs, they should be discontinued and replaced promptly. In emergency situations, such as pre-eclampsia, intravenous labetalol is recommended but intravenous nitroprusside or nitroglycerin could be alternatives. After delivery, BP should be controlled below 140/90 mmHg.

Gestational HTN and preeclampsia are associated with an increased risk of developing HTN after delivery and pre-eclampsia is a risk factor for CVD. Patients with a history of pre-eclampsia have about a two-fold risk of ischemic heart disease, stroke, and venous thrombosis and a four-fold risk of developing sustained HTN. Especially in cases of preeclampsia within 32 weeks of gestation, stillbirth, and fetal growth retardation, the risk of HTN increases much more. Thus, active BP control and lifestyle modification even after delivery is strongly recommended for patients with HTN during pregnancy.

### Women and hypertension

In younger populations, women have lower prevalence of HTN than men. But after menopause, the prevalence of HTN in women increases rapidly as reaches the prevalence in men in their 60s and prevalence becomes even higher than that in men in their 70s or 80s. As for age, the increase in pulse pressure is the same between men and women, however, the systolic and diastolic BPs are higher in women after menopause. Care should be taken in the diagnosis of HTN in menopausal women, because white coat HTN occurs more frequently in these women.

After menopause, weight gain, hormonal changes, and psychological changes occur [171]. In particular, the deficiency of estrogen induces menopausal symptoms and CV events [172]. In the past, hormone replacement therapy (HRT) was widely recommended after menopause, but clinical studies found no beneficial effects for CVD or it sometimes worsened, and therefore, HRT is no longer recommended. Because HRT can increase BP, women who have a greater chance of developing HTN need to be carefully observed for a few months [173].

There is no difference in HTN treatment between women and men. Additionally, there is no difference in BP reduction and drug effects between women and men [174]. Oral contraceptives can increase BP in some subjects, and the occurrence of accelerated or malignant HTN is rare. Family history of HTN, past history of HTN during pregnancy, kidney disease, obesity, or oral contraceptive use can increase the risk of BP increase. Therefore, in the early period of oral contraceptive use, BP needs to be carefully monitored, while periodic measurements are recommended thereafter.

### Sleep apnea

**Table:** 

Recommendations	Class	Level	References
Consider continuous positive pressure ventilation for patients with sleep apnea with HTN.	**IIb**	**B**	[175–178]

Continuous positive airway pressure (CPAP) therapy is effective in improving sleep apnea. However, in studies concerning sleep apnea, CPAP was reported cause a 2–3 mmHg decrease of BP, and the effects differed according to adherence, disease severity, and daytime sleepiness [175–177, 179].

### Cognitive impairment

**Table:** 

Recommendations	Class	Level	References
Consider HTN treatment to prevent cognitive dysfunction and dementia in adult hypertensive patients.	**IIa**	**B**	[55, 180–184]

Vascular disease and its related factors are the major risk factors of dementia, including Alzheimer’s disease [185, 186]. HTN is a major risk factor for ischemic microvascular disease and cerebral white matter disease, which can lead to cognitive dysfunction by damaging the cerebral nerve circuit [187–189]. It has been reported that Alzheimer’s dementia and other dementia were less likely to develop when systolic BP was well controlled. Furthermore, it was more effective in preventing dementia when treatment was started individuals in their 50s rather than in those treated in their 60s [185, 190]. In most randomized clinical trials, HTN treatment did not have a negative impact on cognitive dysfunction and dementia. However, results from studies have been inconsistent due to lack of study power, insufficient follow-up duration, and inadequate tools for cognitive evaluation [55, 180–184].

## Data Availability

Not applicable.

## Abbreviations

ABPM: Ambulatory blood pressure monitoring
ACE: Angiotensin converting enzyme
ARB: Angiotensin II receptor blocker
BMI: Body mass index
BP: Blood pressure
CAD: Coronary artery disease
CKD: Chronic kidney disease
CPAP: Continuous positive airway pressure
CT: Computed tomography
CV: Cardiovascular
CVD: Cardiovascular disease
DBP: Diastolic blood pressure
DM: Diabetes mellitus
eGFR: Estimated glomerular filtration rate.
ESRD: End-stage renal disease
HBPM: Home blood pressure monitoring
HDL: High-density lipoprotein
HRT: Hormone replacement therapy
HTN: Hypertension
LVH: Left ventricular hypertrophy
PAD: Peripheral arterial disease
PDE5: Phosphodiesterase-5
SBP: Systolic blood pressure
t-PA: Tissue-plasminogen activator

## References

[1] National Institute for Health and Clinical Excellence. Hypertension (CG127): clinical management of primary hypertension in adults. http://www.nice.org.uk/guidance/CG127. Accessed 27 June 2019.

[2] Clement DL, De Buyzere ML, De Bacquer DA, de Leeuw PW, Duprez DA, Fagard RH (2003). Prognostic value of ambulatory blood-pressure recordings in patients with treated hypertension. N Engl J Med.

[3] Wang YC, Shimbo D, Muntner P, Moran AE, Krakoff LR, Schwartz JE (2017). Prevalence of masked hypertension among US adults with nonelevated clinic blood pressure. Am J Epidemiol.

[4] Banegas JR, Ruilope LM, de la Sierra A, Vinyoles E, Gorostidi M, de la Cruz JJ (2018). Relationship between clinic and ambulatory blood-pressure measurements and mortality. N Engl J Med.

[5] Verdecchia P, Angeli F (2005). The natural history of white-coat hypertension in the long term. Blood Press Monit.

[6] Ohkubo T, Kikuya M, Metoki H, Asayama K, Obara T, Hashimoto J (2005). Prognosis of “masked” hypertension and “white-coat” hypertension detected by 24-h ambulatory blood pressure monitoring 10-year follow-up from the Ohasama study. J Am Coll Cardiol.

[7] Mancia G, Fagard R, Narkiewicz K, Redon J, Zanchetti A, Bohm M (2013). 2013 ESH/ESC guidelines for the management of arterial hypertension: the task force for the management of arterial hypertension of the European Society of Hypertension (ESH) and of the European Society of Cardiology (ESC). J Hypertens.

[8] Asayama K, Thijs L, Li Y, Gu YM, Hara A, Liu YP (2014). Setting thresholds to varying blood pressure monitoring intervals differentially affects risk estimates associated with white-coat and masked hypertension in the population. Hypertension.

[9] Viera AJ, Lin FC, Tuttle LA, Olsson E, Stankevitz K, Girdler SS (2014). Reproducibility of masked hypertension among adults 30 years or older. Blood Press Monit.

[10] Tuomilehto J, Lindstrom J, Eriksson JG, Valle TT, Hamalainen H, Ilanne-Parikka P (2001). Prevention of type 2 diabetes mellitus by changes in lifestyle among subjects with impaired glucose tolerance. N Engl J Med.

[11] Knowler WC, Barrett-Connor E, Fowler SE, Hamman RF, Lachin JM, Walker EA (2002). Reduction in the incidence of type 2 diabetes with lifestyle intervention or metformin. N Engl J Med.

[12] Dagenais GR, Gerstein HC, Holman R, Budaj A, Escalante A, Hedner T (2008). Effects of ramipril and rosiglitazone on cardiovascular and renal outcomes in people with impaired glucose tolerance or impaired fasting glucose: results of the diabetes REduction assessment with ramipril and rosiglitazone medication (DREAM) trial. Diabetes Care.

[13] McMurray JJ, Holman RR, Haffner SM, Bethel MA, Holzhauer B, Hua TA (2010). Effect of valsartan on the incidence of diabetes and cardiovascular events. N Engl J Med.

[14] Lonn EM, Bosch J, Lopez-Jaramillo P, Zhu J, Liu L, Pais P (2016). Blood-pressure lowering in intermediate-risk persons without cardiovascular disease. N Engl J Med.

[15] Alberti KG, Eckel RH, Grundy SM, Zimmet PZ, Cleeman JI, Donato KA (2009). Harmonizing the metabolic syndrome: a joint interim statement of the international diabetes federation task force on epidemiology and prevention; National Heart, Lung, and Blood Institute; American Heart Association; world heart federation; international atherosclerosis society; and International Association for the Study of obesity. Circulation.

[16] Lee SY, Park HS, Kim DJ, Han JH, Kim SM, Cho GJ (2007). Appropriate waist circumference cutoff points for central obesity in Korean adults. Diabetes Res Clin Pract.

[17] Lee SE, Han K, Kang YM, Kim SO, Cho YK, Ko KS (2018). Trends in the prevalence of metabolic syndrome and its components in South Korea: findings from the Korean National Health Insurance Service database (2009-2013). PLoS One.

[18] Cha MJ, Lee HY, Ahn SV, Han KR, Park JB, Lim SJ (2009). Prevalence and clinical characteristics of metabolic syndrome in Korean hypertensive patients. J Korean Soc Hypertens.

[19] Gami AS, Witt BJ, Howard DE, Erwin PJ, Gami LA, Somers VK (2007). Metabolic syndrome and risk of incident cardiovascular events and death: a systematic review and meta-analysis of longitudinal studies. J Am Coll Cardiol.

[20] Ford ES, Li C, Sattar N (2008). Metabolic syndrome and incident diabetes: current state of the evidence. Diabetes Care.

[21] Cheung BM, Wat NM, Man YB, Tam S, Thomas GN, Leung GM (2007). Development of diabetes in Chinese with the metabolic syndrome: a 6-year prospective study. Diabetes Care.

[22] Mukai N, Doi Y, Ninomiya T, Hata J, Yonemoto K, Iwase M (2009). Impact of metabolic syndrome compared with impaired fasting glucose on the development of type 2 diabetes in a general Japanese population: the Hisayama study. Diabetes Care.

[23] Gress TW, Nieto FJ, Shahar E, Wofford MR, Brancati FL (2000). Hypertension and antihypertensive therapy as risk factors for type 2 diabetes mellitus. Atherosclerosis risk in communities study. N Engl J Med.

[24] Weycker D, Nichols GA, O'Keeffe-Rosetti M, Edelsberg J, Vincze G, Khan ZM (2009). Excess risk of diabetes in persons with hypertension. J Diabetes Complicat.

[25] Emdin CA, Rahimi K, Neal B, Callender T, Perkovic V, Patel A (2015). Blood pressure lowering in type 2 diabetes: a systematic review and meta-analysis. JAMA.

[26] UK Prospective Diabetes Study Group (1998). Tight blood pressure control and risk of macrovascular and microvascular complications in type 2 diabetes: UKPDS 38. BMJ.

[27] Brunstrom M, Carlberg B (2016). Effect of antihypertensive treatment at different blood pressure levels in patients with diabetes mellitus: systematic review and meta-analyses. BMJ.

[28] Cushman WC, Evans GW, Byington RP, Goff DC, Grimm RH, Cutler JA (2010). Effects of intensive blood-pressure control in type 2 diabetes mellitus. N Engl J Med.

[29] Patel A, MacMahon S, Chalmers J, Neal B, Woodward M, Billot L (2007). Effects of a fixed combination of perindopril and indapamide on macrovascular and microvascular outcomes in patients with type 2 diabetes mellitus (the ADVANCE trial): a randomised controlled trial. Lancet.

[30] Group TG (1997). Randomised placebo-controlled trial of effect of ramipril on decline in glomerular filtration rate and risk of terminal renal failure in proteinuric, non-diabetic nephropathy. The GISEN group (Gruppo Italiano di Studi Epidemiologici in Nefrologia). Lancet.

[31] Turnbull F, Neal B, Algert C, Chalmers J, Chapman N, Cutler J (2005). Effects of different blood pressure-lowering regimens on major cardiovascular events in individuals with and without diabetes mellitus: results of prospectively designed overviews of randomized trials. Arch Intern Med.

[32] Turnbull F (2003). Blood pressure lowering treatment Trialists C. effects of different blood-pressure-lowering regimens on major cardiovascular events: results of prospectively-designed overviews of randomised trials. Lancet.

[33] Buckley LF, Dixon DL, GFt W, Wijesinghe DS, Baker WL, Van Tassell BW (2017). Intensive versus standard blood pressure control in SPRINT-eligible participants of ACCORD-BP. Diabetes Care.

[34] Lindholm LH, Ibsen H, Dahlof B, Devereux RB, Beevers G, de Faire U (2002). Cardiovascular morbidity and mortality in patients with diabetes in the losartan intervention for endpoint reduction in hypertension study (LIFE): a randomised trial against atenolol. Lancet.

[35] Berl T, Hunsicker LG, Lewis JB, Pfeffer MA, Porush JG, Rouleau JL (2003). Cardiovascular outcomes in the Irbesartan diabetic nephropathy trial of patients with type 2 diabetes and overt nephropathy. Ann Intern Med.

[36] Palmer SC, Mavridis D, Navarese E, Craig JC, Tonelli M, Salanti G (2015). Comparative efficacy and safety of blood pressure-lowering agents in adults with diabetes and kidney disease: a network meta-analysis. Lancet.

[37] Grundy SM, Benjamin IJ, Burke GL, Chait A, Eckel RH, Howard BV (1999). Diabetes and cardiovascular disease: a statement for healthcare professionals from the American Heart Association. Circulation.

[38] Sowers JR, Haffner S (2002). Treatment of cardiovascular and renal risk factors in the diabetic hypertensive. Hypertension.

[39] Adler AI, Stratton IM, Neil HA, Yudkin JS, Matthews DR, Cull CA (2000). Association of systolic blood pressure with macrovascular and microvascular complications of type 2 diabetes (UKPDS 36): prospective observational study. BMJ.

[40] Hansson L, Zanchetti A, Carruthers SG, Dahlof B, Elmfeldt D, Julius S (1998). Effects of intensive blood-pressure lowering and low-dose aspirin in patients with hypertension: principal results of the hypertension optimal treatment (HOT) randomised trial. HOT study group. Lancet.

[41] Yusuf S, Sleight P, Pogue J, Bosch J, Davies R, Dagenais G (2000). Effects of an angiotensin-converting-enzyme inhibitor, ramipril, on cardiovascular events in high-risk patients. The heart outcomes prevention evaluation study investigators. N Engl J Med.

[42] ALLHAT Officers (2002). Coordinators for the ALLHAT collaborative research group. Major outcomes in high-risk hypertensive patients randomized to angiotensin-converting enzyme inhibitor or calcium channel blocker vs diuretic: the antihypertensive and lipid-lowering treatment to prevent heart attack trial (ALLHAT). JAMA.

[43] Beulens JW, Patel A, Vingerling JR, Cruickshank JK, Hughes AD, Stanton A (2009). Effects of blood pressure lowering and intensive glucose control on the incidence and progression of retinopathy in patients with type 2 diabetes mellitus: a randomised controlled trial. Diabetologia.

[44] Pepine CJ, Handberg EM, Cooper-DeHoff RM, Marks RG, Kowey P, Messerli FH (2003). A calcium channel blocker vs a non-calcium channel blocker hypertension treatment strategy for patients with coronary artery disease. The international verapamil-Trandolapril study (INVEST): a randomized controlled trial. JAMA.

[45] Weber MA, Mansfield TA, Alessi F, Iqbal N, Parikh S, Ptaszynska A (2016). Effects of dapagliflozin on blood pressure in hypertensive diabetic patients on renin-angiotensin system blockade. Blood Press.

[46] Weber MA, Mansfield TA, Cain VA, Iqbal N, Parikh S, Ptaszynska A (2016). Blood pressure and glycaemic effects of dapagliflozin versus placebo in patients with type 2 diabetes on combination antihypertensive therapy: a randomised, double-blind, placebo-controlled, phase 3 study. Lancet Diabetes Endocrinol.

[47] Scheen AJ (2016). Effects of reducing blood pressure on cardiovascular outcomes and mortality in patients with type 2 diabetes: focus on SGLT2 inhibitors and EMPA-REG OUTCOME. Diabetes Res Clin Pract.

[48] Williamson JD, Supiano MA, Applegate WB, Berlowitz DR, Campbell RC, Chertow GM (2016). Intensive vs standard blood pressure control and cardiovascular disease outcomes in adults aged >/=75 years: a randomized clinical trial. JAMA.

[49] Weiss J, Freeman M, Low A, Fu R, Kerfoot A, Paynter R (2017). Benefits and harms of intensive blood pressure treatment in adults aged 60 years or older: a systematic review and meta-analysis. Ann Intern Med.

[50] Whelton PK, Appel LJ, Espeland MA, Applegate WB, Ettinger WH, Kostis JB (1998). Sodium reduction and weight loss in the treatment of hypertension in older persons: a randomized controlled trial of nonpharmacologic interventions in the elderly (TONE). TONE collaborative research group. JAMA.

[51] Rakugi H, Ogihara T, Goto Y, Ishii M, Group JS (2010). Comparison of strict- and mild-blood pressure control in elderly hypertensive patients: a per-protocol analysis of JATOS. Hypertens Res.

[52] Prevention of stroke by antihypertensive drug treatment in older persons with isolated systolic hypertension. Final results of the Systolic Hypertension in the Elderly Program (SHEP). SHEP Cooperative Research Group. Jama. 1991;265:3255–64. 2046107

[53] Staessen JA, Fagard R, Thijs L, Celis H, Arabidze GG, Birkenhager WH (1997). Randomised double-blind comparison of placebo and active treatment for older patients with isolated systolic hypertension. The systolic hypertension in Europe (Syst-Eur) trial investigators. Lancet.

[54] Wing LM, Reid CM, Ryan P, Beilin LJ, Brown MA, Jennings GL (2003). A comparison of outcomes with angiotensin-converting--enzyme inhibitors and diuretics for hypertension in the elderly. N Engl J Med.

[55] Lithell H, Hansson L, Skoog I, Elmfeldt D, Hofman A, Olofsson B (2003). The study on cognition and prognosis in the elderly (SCOPE): principal results of a randomized double-blind intervention trial. J Hypertens.

[56] Saiz LC, Gorricho J, Garjon J, Celaya MC, Muruzabal L, Malon MDM (2017). Blood pressure targets for the treatment of people with hypertension and cardiovascular disease. Cochrane Database Syst Rev.

[57] Mancia G, Kjeldsen SE, Zappe DH, Holzhauer B, Hua TA, Zanchetti A (2016). Cardiovascular outcomes at different on-treatment blood pressures in the hypertensive patients of the VALUE trial. Eur Heart J.

[58] Bangalore S, Kumar S, Volodarskiy A, Messerli FH (2013). Blood pressure targets in patients with coronary artery disease: observations from traditional and Bayesian random effects meta-analysis of randomised trials. Heart.

[59] Cooper-DeHoff RM, Gong Y, Handberg EM, Bavry AA, Denardo SJ, Bakris GL (2010). Tight blood pressure control and cardiovascular outcomes among hypertensive patients with diabetes and coronary artery disease. JAMA.

[60] Group SR (2015). Wright JT, Jr., Williamson JD, Whelton PK, Snyder JK, Sink KM, et al. a randomized trial of intensive versus standard blood-pressure control. N Engl J Med.

[61] Lewington S, Clarke R, Qizilbash N, Peto R, Collins R, Prospective SC (2002). Age-specific relevance of usual blood pressure to vascular mortality: a meta-analysis of individual data for one million adults in 61 prospective studies. Lancet.

[62] Mancia G, Messerli F, Bakris G, Zhou Q, Champion A, Pepine CJ (2007). Blood pressure control and improved cardiovascular outcomes in the international verapamil SR-Trandolapril study. Hypertension.

[63] Law MR, Morris JK, Wald NJ (2009). Use of blood pressure lowering drugs in the prevention of cardiovascular disease: meta-analysis of 147 randomised trials in the context of expectations from prospective epidemiological studies. BMJ.

[64] Borghi C, Bacchelli S, Esposti DD, Bignamini A, Magnani B, Ambrosioni E (1999). Effects of the administration of an angiotensin-converting enzyme inhibitor during the acute phase of myocardial infarction in patients with arterial hypertension. SMILE study investigators. Survival of myocardial infarction long-term evaluation. Am J Hypertens.

[65] Yancy CW, Jessup M, Bozkurt B, Butler J, Casey DE, Colvin MM (2017). 2017 ACC/AHA/HFSA focused update of the 2013 ACCF/AHA guideline for the Management of Heart Failure: a report of the American College of Cardiology/American Heart Association task force on clinical practice guidelines and the Heart Failure Society of America. Circulation.

[66] Lee SE, Lee HY, Cho HJ, Choe WS, Kim H, Choi JO (2017). Reverse J-curve relationship between on-treatment blood pressure and mortality in patients with heart failure. JACC Heart Fail.

[67] Tocci G, Sciarretta S, Volpe M (2008). Development of heart failure in recent hypertension trials. J Hypertens.

[68] Gottdiener JS, Arnold AM, Aurigemma GP, Polak JF, Tracy RP, Kitzman DW (2000). Predictors of congestive heart failure in the elderly: the cardiovascular health study. J Am Coll Cardiol.

[69] Levy D, Larson MG, Vasan RS, Kannel WB, Ho KK (1996). The progression from hypertension to congestive heart failure. JAMA.

[70] Yancy CW, Jessup M, Bozkurt B, Butler J, Casey DE, Writing Committee M (2016). 2016 ACC/AHA/HFSA focused update on new pharmacological therapy for heart failure: an update of the 2013 ACCF/AHA guideline for the Management of Heart Failure: a report of the American College of Cardiology/American Heart Association task force on clinical practice guidelines and the Heart Failure Society of America. Circulation.

[71] Goldstein RE, Boccuzzi SJ, Cruess D, Nattel S (1991). Diltiazem increases late-onset congestive heart failure in postinfarction patients with early reduction in ejection fraction. The adverse experience committee; and the multicenter diltiazem Postinfarction research group. Circulation.

[72] Camm AJ, Kirchhof P, Lip GY, Schotten U, Savelieva I, Ernst S (2010). European heart rhythm, association. European Association for Cardio-Thoracic, surgery. Guidelines for the management of atrial fibrillation: the task force for the Management of Atrial Fibrillation of the European Society of Cardiology (ESC). Eur Heart J.

[73] Kirchhof P, Lip GY, Van Gelder IC, Bax J, Hylek E, Kaab S (2011). Comprehensive risk reduction in patients with atrial fibrillation: emerging diagnostic and therapeutic options. Executive summary of the report from the 3rd AFNET/EHRA consensus conference. Thromb Haemost.

[74] Camm AJ, Lip GY, De Caterina R, Savelieva I, Atar D, Hohnloser SH (2012). 2012 focused update of the ESC guidelines for the management of atrial fibrillation: an update of the 2010 ESC guidelines for the management of atrial fibrillation. Developed with the special contribution of the European heart rhythm association. Eur Heart J.

[75] Manolis AJ, Rosei EA, Coca A, Cifkova R, Erdine SE, Kjeldsen S (2012). Hypertension and atrial fibrillation: diagnostic approach, prevention and treatment. Position paper of the working group ‘Hypertension arrhythmias and Thrombosis’ of the European Society of Hypertension. J Hypertens.

[76] Arima H, Anderson C, Omae T, Woodward M, MacMahon S, Mancia G (2012). Effects of blood pressure lowering on intracranial and extracranial bleeding in patients on antithrombotic therapy: the PROGRESS trial. Stroke.

[77] Vidal-Petiot E, Ford I, Greenlaw N, Ferrari R, Fox KM, Tardif JC (2016). Cardiovascular event rates and mortality according to achieved systolic and diastolic blood pressure in patients with stable coronary artery disease: an international cohort study. Lancet.

[78] Vemulapalli S, Hellkamp AS, Jones WS, Piccini JP, Mahaffey KW, Becker RC (2016). Blood pressure control and stroke or bleeding risk in anticoagulated patients with atrial fibrillation: results from the ROCKET AF trial. Am Heart J.

[79] Zanchetti A, Bond MG, Hennig M, Neiss A, Mancia G, Dal Palu C (2002). Calcium channel blocker lacidipine slows down progression of asymptomatic carotid atherosclerosis: principal results of the European Lacidipine study on atherosclerosis (ELSA), a randomized, double-blind, long-term trial. Circulation.

[80] Zanchetti A, Crepaldi G, Bond MG, Gallus G, Veglia F, Mancia G (2004). Different effects of antihypertensive regimens based on fosinopril or hydrochlorothiazide with or without lipid lowering by pravastatin on progression of asymptomatic carotid atherosclerosis: principal results of PHYLLIS--a randomized double-blind trial. Stroke.

[81] Yen CH, Lai YH, Hung CL, Lee PY, Kuo JY, Yeh HI (2014). Angiotensin receptor blockades effect on peripheral muscular and central aortic arterial stiffness: a meta-analysis of randomized controlled trials and systematic review. Acta Cardiol Sin.

[82] Chen X, Huang B, Liu M, Li X (2015). Effects of different types of antihypertensive agents on arterial stiffness: a systematic review and meta-analysis of randomized controlled trials. J Thorac Dis.

[83] Boutouyrie P, Achouba A, Trunet P, Laurent S, Group ET (2010). Amlodipine-valsartan combination decreases central systolic blood pressure more effectively than the amlodipine-atenolol combination: the EXPLOR study. Hypertension.

[84] Ong KT, Delerme S, Pannier B, Safar ME, Benetos A, Laurent S (2011). Aortic stiffness is reduced beyond blood pressure lowering by short-term and long-term antihypertensive treatment: a meta-analysis of individual data in 294 patients. J Hypertens.

[85] Shahin Y, Khan JA, Chetter I (2012). Angiotensin converting enzyme inhibitors effect on arterial stiffness and wave reflections: a meta-analysis and meta-regression of randomised controlled trials. Atherosclerosis.

[86] Karalliedde J, Smith A, DeAngelis L, Mirenda V, Kandra A, Botha J (2008). Valsartan improves arterial stiffness in type 2 diabetes independently of blood pressure lowering. Hypertension.

[87] Guerin AP, Blacher J, Pannier B, Marchais SJ, Safar ME, London GM (2001). Impact of aortic stiffness attenuation on survival of patients in end-stage renal failure. Circulation.

[88] Murabito JM, Evans JC, D'Agostino RB, Wilson PW, Kannel WB (2005). Temporal trends in the incidence of intermittent claudication from 1950 to 1999. Am J Epidemiol.

[89] Aboyans V, Ricco JB, Bartelink MEL, Bjorck M, Brodmann M, Cohnert T (2018). 2017 ESC guidelines on the diagnosis and treatment of peripheral arterial diseases, in collaboration with the European Society for Vascular Surgery (ESVS): document covering atherosclerotic disease of extracranial carotid and vertebral, mesenteric, renal, upper and lower extremity arteriesEndorsed by: the European stroke organization (ESO)the task force for the diagnosis and treatment of peripheral arterial diseases of the European Society of Cardiology (ESC) and of the European Society for Vascular Surgery (ESVS). Eur Heart J.

[90] Bavry AA, Anderson RD, Gong Y, Denardo SJ, Cooper-Dehoff RM, Handberg EM (2010). Outcomes among hypertensive patients with concomitant peripheral and coronary artery disease: findings from the INternational VErapamil-SR/Trandolapril STudy. Hypertension.

[91] Thomopoulos C, Parati G, Zanchetti A (2016). Effects of blood pressure lowering on outcome incidence in hypertension: 7. Effects of more vs. less intensive blood pressure lowering and different achieved blood pressure levels - updated overview and meta-analyses of randomized trials. J Hypertens.

[92] Yusuf S, Teo KK, Pogue J, Dyal L, Copland I, Schumacher H (2008). Telmisartan, ramipril, or both in patients at high risk for vascular events. N Engl J Med.

[93] Ostergren J, Sleight P, Dagenais G, Danisa K, Bosch J, Qilong Y (2004). Impact of ramipril in patients with evidence of clinical or subclinical peripheral arterial disease. Eur Heart J.

[94] De Buyzere ML, Clement DL (2008). Management of hypertension in peripheral arterial disease. Prog Cardiovasc Dis.

[95] Zanchetti A, Julius S, Kjeldsen S, McInnes GT, Hua T, Weber M (2006). Outcomes in subgroups of hypertensive patients treated with regimens based on valsartan and amlodipine: an analysis of findings from the VALUE trial. J Hypertens.

[96] Piller LB, Simpson LM, Baraniuk S, Habib GB, Rahman M, Basile JN (2014). Characteristics and long-term follow-up of participants with peripheral arterial disease during ALLHAT. J Gen Intern Med.

[97] Thompson AM, Hu T, Eshelbrenner CL, Reynolds K, He J, Bazzano LA (2011). Antihypertensive treatment and secondary prevention of cardiovascular disease events among persons without hypertension: a meta-analysis. JAMA.

[98] Paravastu SC, Mendonca DA, da Silva A (2009). Beta blockers for peripheral arterial disease. Eur J Vasc Endovasc Surg.

[99] Radack K, Deck C (1991). Beta-adrenergic blocker therapy does not worsen intermittent claudication in subjects with peripheral arterial disease. A meta-analysis of randomized controlled trials. Arch Intern Med.

[100] Singer DR, Kite A (2008). Management of hypertension in peripheral arterial disease: does the choice of drugs matter?. Eur J Vasc Endovasc Surg.

[101] Shores J, Berger KR, Murphy EA, Pyeritz RE (1994). Progression of aortic dilatation and the benefit of long-term beta-adrenergic blockade in Marfan's syndrome. N Engl J Med.

[102] Lewis EJ, Hunsicker LG, Clarke WR, Berl T, Pohl MA, Lewis JB (2001). Renoprotective effect of the angiotensin-receptor antagonist irbesartan in patients with nephropathy due to type 2 diabetes. N Engl J Med.

[103] Jafar TH, Schmid CH, Landa M, Giatras I, Toto R, Remuzzi G (2001). Angiotensin-converting enzyme inhibitors and progression of nondiabetic renal disease. A meta-analysis of patient-level data. Ann Intern Med.

[104] Brenner BM, Cooper ME, de Zeeuw D, Keane WF, Mitch WE, Parving HH (2001). Effects of losartan on renal and cardiovascular outcomes in patients with type 2 diabetes and nephropathy. N Engl J Med.

[105] Kim S, Song YR, Chin HJ, Oh YK, Oh KH, Joo KW (2008). The prevalence of chronic kidney disease and the predictors of decreased kidney function in hypertensive patients. Korean J Nephrol.

[106] Kim YJ, Kwak C (2011). Prevalence and associated risk factors for cardiovascular disease: findings from the 2005, 2007 Korea National Health and nutrition examination survey. Korean J Health Promot.

[107] Wheeler DC, Becker GJ (2013). Summary of KDIGO guideline. What do we really know about management of blood pressure in patients with chronic kidney disease?. Kidney Int.

[108] Whelton PK, Carey RM, Aronow WS, Casey DE Jr, Collins KJ, Dennison Himmelfarb C, et al. 2017 ACC/AHA/AAPA/ABC/ACPM/AGS/APhA/ASH/ASPC/NMA/PCNA guideline for the prevention, detection, evaluation, and Management of High Blood Pressure in adults: a report of the American College of Cardiology/American Heart Association task force on clinical practice guidelines. Hypertension. 2018;71:e13–e115. 10.1161/HYP.0000000000000065 29133356

[109] Peterson JC, Adler S, Burkart JM, Greene T, Hebert LA, Hunsicker LG (1995). Blood pressure control, proteinuria, and the progression of renal disease. The modification of diet in renal disease study. Ann Intern Med.

[110] Wright JT, Bakris G, Greene T, Agodoa LY, Appel LJ, Charleston J (2002). Effect of blood pressure lowering and antihypertensive drug class on progression of hypertensive kidney disease: results from the AASK trial. JAMA.

[111] Ruggenenti P, Perna A, Loriga G, Ganeva M, Ene-Iordache B, Turturro M (2005). Blood-pressure control for renoprotection in patients with non-diabetic chronic renal disease (REIN-2): multicentre, randomised controlled trial. Lancet.

[112] Jafar TH, Stark PC, Schmid CH, Landa M, Maschio G, de Jong PE (2003). Progression of chronic kidney disease: the role of blood pressure control, proteinuria, and angiotensin-converting enzyme inhibition: a patient-level meta-analysis. Ann Intern Med.

[113] Maschio G, Alberti D, Janin G, Locatelli F, Mann JF, Motolese M (1996). Effect of the angiotensin-converting-enzyme inhibitor benazepril on the progression of chronic renal insufficiency. The angiotensin-converting-enzyme inhibition in progressive renal insufficiency study group. N Engl J Med.

[114] Ruggenenti P, Perna A, Benini R, Bertani T, Zoccali C, Maggiore Q (1999). In chronic nephropathies prolonged ACE inhibition can induce remission: dynamics of time-dependent changes in GFR. Investigators of the GISEN group. Gruppo Italiano Studi Epidemiologici in Nefrologia. J Am Soc Nephrol.

[115] Krikken JA, Laverman GD, Navis G (2009). Benefits of dietary sodium restriction in the management of chronic kidney disease. Curr Opin Nephrol Hypertens.

[116] Boudville N, Ward S, Benaroia M, House AA (2005). Increased sodium intake correlates with greater use of antihypertensive agents by subjects with chronic kidney disease. Am J Hypertens.

[117] Gelber RP, Kurth T, Kausz AT, Manson JE, Buring JE, Levey AS (2005). Association between body mass index and CKD in apparently healthy men. Am J Kidney Dis.

[118] Jones DW, Kim JS, Andrew ME, Kim SJ, Hong YP (1994). Body mass index and blood pressure in Korean men and women: the Korean National Blood Pressure Survey. J Hypertens.

[119] Whelton SP, Chin A, Xin X, He J (2002). Effect of aerobic exercise on blood pressure: a meta-analysis of randomized, controlled trials. Ann Intern Med.

[120] Kosmadakis GC, John SG, Clapp EL, Viana JL, Smith AC, Bishop NC (2012). Benefits of regular walking exercise in advanced pre-dialysis chronic kidney disease. Nephrol Dial Transplant.

[121] Chen L, Davey Smith G, Harbord RM, Lewis SJ (2008). Alcohol intake and blood pressure: a systematic review implementing a Mendelian randomization approach. PLoS Med.

[122] Kim Yoon Ji, Hwang Seun Deuk, Oh Tae Jung, Kim Kyoung Min, Jang Hak Chul, Kimm Heejin, Kim Hyeon Chang, Jee Sun Ha, Lim Soo (2017). Association Between Obesity and Chronic Kidney Disease, Defined by Both Glomerular Filtration Rate and Albuminuria, in Korean Adults. Metabolic Syndrome and Related Disorders.

[123] Acelajado MC, Oparil S (2009). Hypertension in the elderly. Clin Geriatr Med.

[124] Benvenuto LJ, Krakoff LR (2011). Morbidity and mortality of orthostatic hypotension: implications for management of cardiovascular disease. Am J Hypertens.

[125] Wu JS, Yang YC, Lu FH, Wu CH, Chang CJ (2008). Population-based study on the prevalence and correlates of orthostatic hypotension/hypertension and orthostatic dizziness. Hypertens Res.

[126] Lawes CM, Bennett DA, Feigin VL, Rodgers A (2004). Blood pressure and stroke: an overview of published reviews. Stroke.

[127] Psaty BM, Lumley T, Furberg CD, Schellenbaum G, Pahor M, Alderman MH (2003). Health outcomes associated with various antihypertensive therapies used as first-line agents: a network meta-analysis. JAMA.

[128] Wiysonge CSBH, Mayosi BM, Maroney R, Mbewu A, Opie LH, Volmink J (2007). Beta-blockers for hypertension.

[129] Aronow WS, Frishman WH (2004). Treatment of hypertension and prevention of ischemic stroke. Curr Cardiol Rep.

[130] National Institute of Neurological D, Stroke rt PASSG (1995). Tissue plasminogen activator for acute ischemic stroke. N Engl J Med.

[131] Hacke W, Kaste M, Bluhmki E, Brozman M, Davalos A, Guidetti D (2008). Thrombolysis with alteplase 3 to 4.5 hours after acute ischemic stroke. N Engl J Med.

[132] Ahmed N, Wahlgren N, Brainin M, Castillo J, Ford GA, Kaste M (2009). Relationship of blood pressure, antihypertensive therapy, and outcome in ischemic stroke treated with intravenous thrombolysis: retrospective analysis from safe implementation of thrombolysis in stroke-international stroke thrombolysis register (SITS-ISTR). Stroke.

[133] Robinson TG, Potter JF, Ford GA, Bulpitt CJ, Chernova J, Jagger C (2010). Effects of antihypertensive treatment after acute stroke in the continue or stop post-stroke Antihypertensives collaborative study (COSSACS): a prospective, randomised, open, blinded-endpoint trial. Lancet Neurol.

[134] He J, Zhang Y, Xu T, Zhao Q, Wang D, Chen CS (2014). Effects of immediate blood pressure reduction on death and major disability in patients with acute ischemic stroke: the CATIS randomized clinical trial. JAMA.

[135] Chobanian AV, Bakris GL, Black HR, Cushman WC, Green LA, Izzo JL (2003). Seventh report of the joint National Committee on prevention, detection, evaluation, and treatment of high blood pressure. Hypertension.

[136] Sandset EC, Bath PM, Boysen G, Jatuzis D, Korv J, Luders S (2011). The angiotensin-receptor blocker candesartan for treatment of acute stroke (SCAST): a randomised, placebo-controlled, double-blind trial. Lancet.

[137] Qureshi AI, Ezzeddine MA, Nasar A, Suri MF, Kirmani JF, Hussein HM (2007). Prevalence of elevated blood pressure in 563,704 adult patients with stroke presenting to the ED in the United States. Am J Emerg Med.

[138] Schrader J, Luders S, Kulschewski A, Berger J, Zidek W, Treib J (2003). The ACCESS study: evaluation of acute candesartan Cilexetil therapy in stroke survivors. Stroke.

[139] Hankey GJ (2011). Lowering blood pressure in acute stroke: the SCAST trial. Lancet.

[140] Jauch EC, Saver JL, Adams HP, Bruno A, Connors JJ, Demaerschalk BM (2013). Guidelines for the early management of patients with acute ischemic stroke: a guideline for healthcare professionals from the American Heart Association/American Stroke Association. Stroke.

[141] Clinical Practice Guidelines for Stroke. http://www.stroke.or.kr/eng//image/main/CPGStrok(English)20130730.pdf. Accessed 11 Oct 2018.

[142] Larrue V, von Kummer R, del Zoppo G, Bluhmki E (1997). Hemorrhagic transformation in acute ischemic stroke. Potential contributing factors in the European cooperative acute stroke study. Stroke.

[143] Intracerebral hemorrhage after intravenous t-PA therapy for ischemic stroke. The NINDS t-PA Stroke Study Group. Stroke. 1997;28:2109–18. 10.1161/01.str.28.11.2109 9368550

[144] Adams HP, del Zoppo G, Alberts MJ, Bhatt DL, Brass L, Furlan A (2007). Guidelines for the early management of adults with ischemic stroke: a guideline from the American Heart Association/American Stroke Association Stroke Council, Clinical Cardiology Council, Cardiovascular Radiology and Intervention Council, and the Atherosclerotic Peripheral Vascular Disease and Quality of Care Outcomes in Research Interdisciplinary Working Groups: the American Academy of Neurology affirms the value of this guideline as an educational tool for neurologists. Stroke.

[145] Anderson CS, Heeley E, Huang Y, Wang J, Stapf C, Delcourt C (2013). Rapid blood-pressure lowering in patients with acute intracerebral hemorrhage. N Engl J Med.

[146] Qureshi AI, Palesch YY, Barsan WG, Hanley DF, Hsu CY, Martin RL (2016). Intensive blood-pressure lowering in patients with acute cerebral hemorrhage. N Engl J Med.

[147] Rodriguez-Luna D, Pineiro S, Rubiera M, Ribo M, Coscojuela P, Pagola J (2013). Impact of blood pressure changes and course on hematoma growth in acute intracerebral hemorrhage. Eur J Neurol.

[148] Sakamoto Y, Koga M, Yamagami H, Okuda S, Okada Y, Kimura K (2013). Systolic blood pressure after intravenous antihypertensive treatment and clinical outcomes in hyperacute intracerebral hemorrhage: the stroke acute management with urgent risk-factor assessment and improvement-intracerebral hemorrhage study. Stroke.

[149] Tsivgoulis G, Katsanos AH, Butcher KS, Boviatsis E, Triantafyllou N, Rizos I (2014). Intensive blood pressure reduction in acute intracerebral hemorrhage: a meta-analysis. Neurology.

[150] Ohwaki K, Yano E, Nagashima H, Hirata M, Nakagomi T, Tamura A (2004). Blood pressure management in acute intracerebral hemorrhage: relationship between elevated blood pressure and hematoma enlargement. Stroke.

[151] Morgenstern LB, Hemphill JC, Anderson C, Becker K, Broderick JP, Connolly ES (2010). Guidelines for the management of spontaneous intracerebral hemorrhage: a guideline for healthcare professionals from the American Heart Association/American Stroke Association. Stroke.

[152] Liu L, Wang Z, Gong L, Zhang Y, Thijs L, Staessen JA (2009). Blood pressure reduction for the secondary prevention of stroke: a Chinese trial and a systematic review of the literature. Hypertens Res.

[153] Lakhan SE, Sapko MT (2009). Blood pressure lowering treatment for preventing stroke recurrence: a systematic review and meta-analysis. Int Arch Med.

[154] Progress Collaborative Group (2001). Randomised trial of a perindopril-based blood-pressure-lowering regimen among 6,105 individuals with previous stroke or transient ischaemic attack. Lancet.

[155] Chen GJ, Yang MS (2013). The effects of calcium channel blockers in the prevention of stroke in adults with hypertension: a meta-analysis of data from 273,543 participants in 31 randomized controlled trials. PLoS One.

[156] Ettehad D, Emdin CA, Kiran A, Anderson SG, Callender T, Emberson J (2016). Blood pressure lowering for prevention of cardiovascular disease and death: a systematic review and meta-analysis. Lancet.

[157] Arima H, Chalmers J, Woodward M, Anderson C, Rodgers A, Davis S (2006). Lower target blood pressures are safe and effective for the prevention of recurrent stroke: the PROGRESS trial. J Hypertens.

[158] Benavente OR, Coffey CS, Conwit R, Hart RG, McClure LA, Group SPSS (2013). Blood-pressure targets in patients with recent lacunar stroke: the SPS3 randomised trial. Lancet.

[159] Rashid P, Leonardi-Bee J, Bath P (2003). Blood pressure reduction and secondary prevention of stroke and other vascular events: a systematic review. Stroke.

[160] PATS Collaborating Group (1995). Poststroke antihypertensive treatment study. A preliminary result. Chin Med J.

[161] Dong JY, Zhang YH, Qin LQ (2011). Erectile dysfunction and risk of cardiovascular disease: meta-analysis of prospective cohort studies. J Am Coll Cardiol.

[162] Scranton RE, Lawler E, Botteman M, Chittamooru S, Gagnon D, Lew R (2007). Effect of treating erectile dysfunction on management of systolic hypertension. Am J Cardiol.

[163] Nunes KP, Labazi H, Webb RC (2012). New insights into hypertension-associated erectile dysfunction. Curr Opin Nephrol Hypertens.

[164] Manolis A, Doumas M (2008). Sexual dysfunction: the ‘prima ballerina’ of hypertension-related quality-of-life complications. J Hypertens.

[165] Pickering TG, Shepherd AM, Puddey I, Glasser DB, Orazem J, Sherman N (2004). Sildenafil citrate for erectile dysfunction in men receiving multiple antihypertensive agents: a randomized controlled trial. Am J Hypertens.

[166] McConnell JD, Roehrborn CG, Bautista OM, Andriole GL, Dixon CM, Kusek JW (2003). The long-term effect of doxazosin, finasteride, and combination therapy on the clinical progression of benign prostatic hyperplasia. N Engl J Med.

[167] Mancia G, De Backer G, Dominiczak A, Cifkova R, Fagard R, Germano G (2007). 2007 guidelines for the management of arterial hypertension: the task force for the management of arterial hypertension of the European Society of Hypertension (ESH) and of the European Society of Cardiology (ESC). Eur Heart J.

[168] Kuklina EV, Tong X, Bansil P, George MG, Callaghan WM (2011). Trends in pregnancy hospitalizations that included a stroke in the United States from 1994 to 2007: reasons for concern?. Stroke.

[169] Regitz-Zagrosek V, Blomstrom Lundqvist C, Borghi C, Cifkova R, Ferreira R, Foidart JM (2011). European society of, gynecology. Association for European Paediatric Cardiology. German Society for Gender Medicine. ESC guidelines on the management of cardiovascular diseases during pregnancy: the task force on the Management of Cardiovascular Diseases during pregnancy of the European Society of Cardiology (ESC). Eur Heart J.

[170] Excellence NIfHaC (2011). Hypertension in pregnancy. The management of hypertensive disorders during pregnancy.

[171] Reckelhoff JF, Fortepiani LA (2004). Novel mechanisms responsible for postmenopausal hypertension. Hypertension.

[172] Staessen JA, Celis H, Fagard R (1998). The epidemiology of the association between hypertension and menopause. J Hum Hypertens.

[173] Chasan-Taber L, Willett WC, Manson JE, Spiegelman D, Hunter DJ, Curhan G (1996). Prospective study of oral contraceptives and hypertension among women in the United States. Circulation.

[174] Turnbull F, Woodward M, Neal B, Barzi F, Ninomiya T, Chalmers J (2008). Do men and women respond differently to blood pressure-lowering treatment? Results of prospectively designed overviews of randomized trials. Eur Heart J.

[175] Barbe F, Duran-Cantolla J, Capote F, de la Pena M, Chiner E, Masa JF (2010). Long-term effect of continuous positive airway pressure in hypertensive patients with sleep apnea. Am J Respir Crit Care Med.

[176] Martinez-Garcia MA, Capote F, Campos-Rodriguez F, Lloberes P, Diaz de Atauri MJ, Somoza M (2013). Effect of CPAP on blood pressure in patients with obstructive sleep apnea and resistant hypertension: the HIPARCO randomized clinical trial. JAMA.

[177] Lozano L, Tovar JL, Sampol G, Romero O, Jurado MJ, Segarra A (2010). Continuous positive airway pressure treatment in sleep apnea patients with resistant hypertension: a randomized, controlled trial. J Hypertens.

[178] Pedrosa RP, Drager LF, de Paula LKG, Amaro ACS, Bortolotto LA, Lorenzi-Filho G (2013). Effects of OSA treatment on BP in patients with resistant hypertension: a randomized trial. Chest.

[179] Pedrosa RP, Drager LF, Gonzaga CC, Sousa MG, de Paula LK, Amaro AC (2011). Obstructive sleep apnea: the most common secondary cause of hypertension associated with resistant hypertension. Hypertension.

[180] Peters R, Beckett N, Forette F, Tuomilehto J, Clarke R, Ritchie C (2008). Incident dementia and blood pressure lowering in the hypertension in the very elderly trial cognitive function assessment (HYVET-COG): a double-blind, placebo controlled trial. Lancet Neurol.

[181] Applegate WB, Pressel S, Wittes J, Luhr J, Shekelle RB, Camel GH (1994). Impact of the treatment of isolated systolic hypertension on behavioral variables. Results from the systolic hypertension in the elderly program. Arch Intern Med.

[182] Forette F, Seux ML, Staessen JA, Thijs L, Birkenhager WH, Babarskiene MR (1998). Prevention of dementia in randomised double-blind placebo-controlled systolic hypertension in Europe (Syst-Eur) trial. Lancet.

[183] Forette F, Seux ML, Staessen JA, Thijs L, Babarskiene MR, Babeanu S (2002). The prevention of dementia with antihypertensive treatment: new evidence from the systolic hypertension in Europe (Syst-Eur) study. Arch Intern Med.

[184] Tzourio C, Anderson C, Chapman N, Woodward M, Neal B, MacMahon S (2003). Effects of blood pressure lowering with perindopril and indapamide therapy on dementia and cognitive decline in patients with cerebrovascular disease. Arch Intern Med.

[185] Qiu C, Winblad B, Fratiglioni L (2005). The age-dependent relation of blood pressure to cognitive function and dementia. Lancet Neurol.

[186] Kuller LH, Lopez OL, Jagust WJ, Becker JT, DeKosky ST, Lyketsos C (2005). Determinants of vascular dementia in the cardiovascular health cognition study. Neurology.

[187] Liao D, Cooper L, Cai J, Toole JF, Bryan NR, Hutchinson RG (1996). Presence and severity of cerebral white matter lesions and hypertension, its treatment, and its control. The ARIC study. Atherosclerosis risk in communities study. Stroke.

[188] Longstreth WT, Manolio TA, Arnold A, Burke GL, Bryan N, Jungreis CA (1996). Clinical correlates of white matter findings on cranial magnetic resonance imaging of 3301 elderly people. The Cardiovascular Health Study. Stroke.

[189] O'Rourke MF, Safar ME (2005). Relationship between aortic stiffening and microvascular disease in brain and kidney: cause and logic of therapy. Hypertension.

[190] Hughes TM, Sink KM (2016). Hypertension and its role in cognitive function: current evidence and challenges for the future. Am J Hypertens.

